# MicroRNAs and SIRT1: A Strategy for Stem Cell Renewal and Clinical Development?

**Published:** 2015-10-08

**Authors:** Kenneth Maiese

**Affiliations:** Cellular and Molecular Signaling, Newark, New Jersey 07101, USA

**Keywords:** Akt, apoptosis, autophagy, forkhead, FoxO, miRNA, mTOR, mTORC1, mTORC2, programmed cell death, small non-coding RNA, sirtuins, SIRT1, stem cells

## Abstract

Small non-coding ribonucleic acids (RNAs), known as microRNAs (miRNAs), are now becoming recognized as significant agents that can affect the onset and progression of numerous disorders throughout the body. In particular, miRNAs also may determine stem cell renewal and differentiation. Intimately tied to the ability of miRNAs to govern stem cell proliferation are the proliferative pathways of silent mating type information regulation 2 homolog 1 (*Saccharomyces cerevisiae*) (SIRT1) and the cell survival mechanisms of autophagy that can be coupled to the activity of the mechanistic target of rapamycin (mTOR). Targeting miRNAs that oversee SIRT1 activity offers interesting prospects for the translation of these pathways into efficacious clinical treatment programs for a host of disorders. Yet, as work in this area progresses, a number of challenges unfold that impact whether manipulation of non-coding RNAs and SIRT1 can finely guide stem cell renewal and differentiation to reach successful clinical outcomes.

## Stem Cell Clinical Utility: Considerations for miRNAs and SIRT1

Stem cells are increasingly being considered for the development of novel strategies for multiple disorders throughout the body that can affect the nervous system, cardiovascular system, immune system, metabolism, and cancer. One of the challenges for applications that rely upon stem cell proliferation and differentiation is the protection and maintenance of stem cell populations. For example, specific pathways, such as the mechanistic target of rapamycin (mTOR), can be critical for stem cell proliferation [1]. In the absence of mTOR activity, trophoblast growth can be inhibited with the failure to establish embryonic stem cells [2]. Loss of mTOR activity in neural stem cells results in reduced lineage expansion and blocked differentiation and neuronal production [3]. During aging, activity of mTOR may be reduced and leads to reduced neurogenesis [4] and a reduction in the proliferation of active neural stem cells [5]. The degree of activity of the mTOR pathway also can impact the differentiation of stem cell populations. Inhibition of mTOR activity can promote cell differentiation into astrocytic cells [6] and lead to earlier neuronal and astroglial differentiation [7]. Furthermore, increased activity of mTOR can foster tumor growth [8,9]. Blockade of mTOR activity can limit the population of cancer stem cells that can cause disease recurrence and therapeutic resistance [10].

Interestingly, loss of mTOR activity can promote the induction of autophagy [11] and lead to an increase in silent mating type information regulation 2 homolog 1 (*Saccharomyces cerevisiae*) (SIRT1) activity that also is vital for stem cell proliferation [12]. In human embryonic stem cells challenged with oxidative stress, autophagy leads to cell protection and requires SIRT1 activity with the concurrent inhibition of mTOR [13]. SIRT1 appears to have an inverse relationship with mTOR to increase stem cell survival [12,14]. During the down-regulation of mTOR, SIRT1 promotes neuronal growth [15] and increases mesangial cell proliferation during high glucose exposure [16]. SIRT1 can limit the expression of aged mesenchymal stem cell phenotypes [17], prevent senescence and impaired differentiation of endothelial progenitor cells [18], and improve cardiomyoblast survival [19]. SIRT1 can influence neuronal differentiation as well. If SIRT1 is repressed with the parallel induction of heat shock protein-70, neural differentiation and the maturation of embryonic cortical neurons can ensue [20]. Differentiation of human embryonic stem cells into motoneurons also occurs in the absence of SIRT1 [21]. As a proliferative agent, increased activity of SIRT1 under some circumstances can lead to the expansion of cancer stem cells. SIRT1 can maintain acute myeloid leukemia stem cells and result in resistance against chemotherapy [22], promote endometrial cell tumor growth through lipogenesis [23], and foster oncogenic transformation of neural stem cells [24].

One strategy that may successfully regulate SIRT1 activity and stem cell proliferation for effective translation into clinical treatment programs may involve the modulation of microRNAs (miRNAs). MiRNAs are composed of 19-25 nucleotides and are small non-coding ribonucleic acids (RNAs). MiRNAs oversee gene expression by silencing targeted messenger RNAs (mRNAs) translated by specific genes. These small non-coding ribonucleic acids may play an important role to influence stem cell proliferation and cellular differentiation. For example, over-expression of miR-381 can lead to neural stem cell proliferation and prevent differentiation into astrocytes [25]. MiR-134, miR-296, and miR-470 can serve to target Oct4, Sox2, and Nanog coding regions to lead to stem cell differentiation [26]. In regards to SIRT1, neuronal differentiation can occur through miR-34a that leads to decreased SIRT1 expression and DNA-binding of p53 in mouse neural stem cells [27]. However, during increased SIRT1 activity, miR-34a results in astrocytic differentiation that appears to be independent of SIRT1 [27]. Under other conditions, a reduction in miRNA activity with increased SIRT1 expression may be necessary for stem cell proliferation. Silencing of miR-195 in old mesenchymal stem cells that allows increased SIRT1 activity restores anti-aging factors expression that include telomerase reverse transcriptase, protein kinase B (Akt), and the forkhead transcription factor FOXO1 [28] to promote stem cell proliferation [29]. In addition, loss of miR-204 that can target SIRT1 can allow SIRT1 to foster the proliferation of spermatogonial stem cells [30]. Given the inverse relationship between mTOR and SIRT1, proliferation of stem cells also may require increased SIRT1 activity in combination with the inhibition or dysfunction of mTOR signaling that is controlled by miRNAs [31].

Targeting miRNAs provides an intriguing format for the control of stem cell proliferation and differentiation through pathways that involve SIRT1. Yet, several considerations must be addressed for the development of novel strategies with stem cells, miRNAs, and SIRT1. For example, the cellular level of activity of SIRT1 that is controlled by miRNAs may present an important caveat for the development of strategies for clinical disorders, since the presence of SIRT1 has the capability to either promote or retard stem cell proliferation and differentiation. To a similar degree, the level of SIRT1 activity can ultimately influence cellular survival. Sufficient SIRT1 activity is required for cellular cardiovascular protection [32-35] and neuronal protection [36-38]. However, a reduction in SIRT1 activity may be necessary for growth factor protection with insulin growth factor-1 [39]. Other considerations involve the role of programmed cell death pathways that involve autophagy or apoptosis as well as mTOR with miRNAs and SIRT1. SIRT1 can promote autophagy induction during inhibition of mTOR activity that may be beneficial to stem cell proliferation. Yet, non-coding mRNAs may block autophagy pathways through SIRT1 and prevent potentially reparative stem cell pathways such as angiogenesis [40]. In addition, some miRNAs, such as miR-34a, have been reported to lead to apoptosis, impaired cell vitality, and aggravated senescence in mesenchymal stem cells through the activation of the SIRT1 and FOXO3a [41], clearly suggesting that SIRT1 activity regulated by miRNAs can greatly affect not only stem cell proliferation and differentiation, but also stem cell survival.
